# Levosimendan protects human hepatocytes from ischemia-reperfusion injury

**DOI:** 10.1371/journal.pone.0187839

**Published:** 2017-11-16

**Authors:** Stefanie N. Brunner, Nicolai V. Bogert, Andreas A. Schnitzbauer, Eva Juengel, Anton Moritz, Isabella Werner, Angela Kornberger, Andres Beiras-Fernandez

**Affiliations:** 1 Department of Thoracic and Cardiovascular Surgery, University Hospital Frankfurt, Goethe University, Frankfurt/Main, Germany; 2 Department of Cardiology, University Hospital Heidelberg, Ruprecht-Karls-University, Heidelberg, Germany; 3 Clinic for General and Visceral Surgery, University Hospital Frankfurt, Goethe University, Frankfurt/Main, Germany; 4 Department of Urology, University Hospital Mainz, Johannes Gutenberg University, Mainz, Germany; 5 Department of Thoracic and Cardiovascular Surgery, University Hospital Mainz, Johannes Gutenberg University, Mainz, Germany; University of PECS Medical School, HUNGARY

## Abstract

**Background:**

Ischemia-reperfusion injury (IRI) is a major challenge in liver transplantation. The mitochondrial pathway plays a pivotal role in hepatic IRI. Levosimendan, a calcium channel sensitizer, was shown to attenuate apoptosis after IRI in animal livers. The aim of this study was to investigate the effect of levosimendan on apoptosis in human hepatocytes.

**Methods:**

Primary human hepatocytes were either exposed to hypoxia or cultured under normoxic conditions. After the hypoxic phase, reoxygenation was implemented and cells were treated with different concentrations of levosimendan (10ng/ml, 100ng/ml, 1000ng/ml). The overall metabolic activity of the cells was measured using 3-(4,5-dimethylthiazol-2-yl)-2,5-diphenyltetrazolium bromide (MTT), and aspartate aminotransferase (AST) levels were determined in order to quantify hepatic injury. Fluorescence-activated cell sorting (FACS) analysis was applied to measure necrosis and apoptosis. Finally, Western blotting was performed to analyze apoptotic pathway proteins.

**Results:**

Administration of levosimendan during reperfusion increases the metabolic activity of human hepatocytes and decreases AST levels. Moreover, apoptosis after IRI is reduced in treated vs. untreated hepatocytes, and levosimendan prevents down-regulation of the anti-apoptotic protein Bcl-2 as well as up-regulation of the pro-apoptotic protein BAX.

**Conclusion:**

The present study suggests a protective effect of levosimendan on human hepatocytes. Our findings suggest that treatment with levosimendan during reperfusion attenuates apoptosis of human hepatocytes by influencing BAX and Bcl-2 levels.

## Introduction

Levosimendan is a substance with positive inotropic and vasodilating properties that is used to treat acute exacerbations of chronic heart failure [1]. Levosimendan increases the calcium sensitivity of cardiac troponin C by stabilizing the Ca-bound conformation of troponin [2] and thereby improves cardiac contractility [3; 4]. In contrast to other inotropic agents, it exerts no effect on the calcium concentration within the cardiac myofilaments and is therefore not associated with increases in myocardial oxygen consumption, which are known to exert potentially deleterious effects in the setting of heart failure. Moreover, it exerts a vasodilatory effect by opening the adenosine triphosphate (ATP)-dependent potassium (K+) channels [5] of coronary smooth muscle cells.

Beyond these cardiac effects, recent animal studies [6] demonstrated anti-apoptotic effects of levosimendan consisting in a modulation of apoptosis achieved by the opening of mitochondrial K(ATP) channels [7]. This causes a stabilization of the mitochondrial inner membrane, thus resulting in a reduction of IRI-related apoptosis.

IRI is a widely distributed phenomenon that occurs wherever tissues are reperfused after ischemia. While ischemia causes metabolic disturbances resulting from a lack of oxygen and adenosine triphosphate, reperfusion is characterized by a severe inflammatory immune response resulting from leukocyte infiltration and release of pro-inflammatory cytokines.

IRI is known to lead to apoptosis in the liver [8] and therefore plays a pivotal role in liver dysfunction after transplantation [9]. Apoptosis, representing a highly regulated process by which cells are eliminated without inflammation [10], may occur through two different pathways. The extrinsic pathway commences with a mediator such as Fas ligand (CD 95) or TNF-α activating a Fas receptor, thus leading to cleavage of pro-caspase 8 into active caspase 8. Caspase 8 can then either directly activate caspase-3 or precipitate the intrinsic pathway by releasing cytochrome c from the mitochondrion into the cytosol [11]. Pro-caspase 9 is then proteolytically activated to caspase 9. The extrinsic and intrinsic pathways both lead up to the same final step that consists of pro-caspase 3 being cleaved into active caspase-3. Caspase-3, finally, causes cell shrinkage and nuclear fragmentation within the apoptotic cell.

The intrinsic pathway additionally comprises proteins from the B-cell lymphoma (Bcl)-2 family. Bcl-2 exerts an anti-apoptotic effect while BAX has pro-apoptotic properties [12]. Both constitute channels within the outer mitochondrial membrane and can either promote or inhibit cytochrome c release [13]. Recent animal studies have shown that levosimendan prevents down-regulation of Bcl-2 [14] and up-regulation of BAX in hepatic cells after IRI [15; 16].

The protective effect of levosimendan in human hepatocytes has not been investigated so far. Hence, in the present study, we investigated the mechanisms underlying the postulated protective effect of levosimendan by examining the roles of BAX and Bcl-2 in human hepatocytes subjected to IRI and treatment with levosimendan.

## Materials and methods

### Experimental design

Human hepatocytes were obtained from life technologies (Carlsbad, California, USA). These cells (HPRGC10) are immortalized human hepatocytes exhibiting many of the characteristics of primary human hepatocytes. For MTT and AST analysis, 5x10^3^ cells/ml were merged and seeded in 96-well plates (Sarstedt, Nümbrecht, Germany) in hepatocyte medium (Promocell, Heidelberg, Germany). The medium used was serum-free.

After an incubation period of 24h, cell viability was tested with tryptophan blue. Subsequently, the cells were divided into two groups (S1 Fig). One group remained under normoxic conditions and was incubated at 37°C in a 5% CO_2_ and 21% O_2_ atmosphere. The cells allocated to the second group (IRI) were incubated at <2% O_2_ for two hours to simulate a period of ischemia. Afterwards, they were reperfused with hepatocyte medium under room air conditions in order to simulate a reperfusion phase. During reperfusion, they were treated with 10ng/ml (D1), 100ng/ml (D2) or 1000 ng/ml (D3) levosimendan (Simdax^®^, Orion Pharma, Sweden) for 24h or 48h. Untreated hepatocytes served as controls.

For FACS and Western blot analysis, cells were treated as described above for MTT and AST but using a 6-well plate (Sarstedt, Nümbrecht, Germany). FACS analysis was implemented after 4h, 8h, 24h, 48h and 72h levosimendan treatment with each of the experiments performed three times. Western blot analysis was applied in the groups treated with 100ng/ml or 1000ng/ml levosimendan for 24h and 48h, with each experiment implemented twice.

#### MTT

The overall metabolic activity of human hepatocytes was measured using the MTT dye reduction assay (Roche Diagnostics, Penzberg, Germany). This assay is based on the reduction of yellow tetrazolium salt (MTT) to purple formazan crystals. This only occurs in the presence of NADH or NADPH, which are only produced by metabolically active cells. The absorbance of the colored solution can be quantified by measuring a certain wavelength using a spectrophotometer.

Human hepatocytes (50μl, 5×10^3^ cells/ml) were seeded onto 96-well culture plates. After 24h and 48htreatment with 1000ng/ml, 100ng/ml and 10ng/ml levosimendan, cells were incubated with MTT (0.5 mg/ml) for 4h. Untreated hepatocytes served as controls, and saponin served as positive control. The cells were then lysed in a solubilisation buffer.

Subsequently, the plates were incubated at 37°C o/n. A microplate reader was used to determine the absorbance at 550 nm for each well. The wavelength was chosen according to the manufacturer’s instructions as well as in accordance with prior tests which had shown maximum absorbance in human hepatocytes at 550 nm.

#### AST

An AST activity assay (Sigma-Aldrich, St. Louis, MO) was used to quantify the release of AST into the cell-free supernatant. Cells were treated with levosimendan as previously described for MTT testing. Following this, the supernatant of each well was separately transferred into 50μl of the reaction mix. The samples were then incubated at 37°C for 2 minutes, and the initial absorbance at 450 nm was measured. Continuing the incubation at 37°C, measuring was repeated every 5 minutes until the value of the most active sample exceeded that of the highest standard. AST levels were presented as percentages of the total activity.

#### FACS

An annexin-V-detection kit (eBioscience, San Diego, CA) was used to assay apoptosis and necrosis of human hepatocytes.

Each group was treated separately. To each FACS tube, 100 μl of cell suspension (1,5x10^6^ cells), 100 μl of staining buffer, 5 μl annexin and propidium iodide were added. Incubation was implemented in accordance with the manufacturer’s instructions.

The percentages of viable, apoptotic (early and late) and necrotic cells were determined on a FACSCantoII flow cytometer. Data were analyzed using FACSDiva 6.1.2 software (BD, Heidelberg, Germany). Cells were merged to exclude cellular debris. Nearly 5000 cells per group were analyzed for apoptotic, necrotic and viable cells. The total amount of cells in the control group was set to 100%. The results of the treatment groups were expressed as proportions of the result in the control group.

#### Western blotting

Protein samples were extracted from human hepatocytes in order to analyze BAX and Bcl-2. For this purpose, the human hepatocytes were lysed and protein concentrations were determined using Coomassie (Thermo Scientific, Waltham, MA). The lysates were centrifuged and proteins were subjected to 7% TGX gel (Biorad, California, 12%) and electrophoresed (30 min, 80V followed by 100V). The proteins were then transferred to nitrocellulose membranes (Biorad, Trans-Blot Turbo Transfer System, 7min). Non-specific binding sites were blocked with 10% non-fat dry milk for 1 hour. The blots were then probed with BAX antibody (dilution 1:5000, abcam, Cambridge, UK) and Bcl-2 antibody (dilution 1:1000, abcam, Cambridge, UK) o/n.

Goat-anti-rabbit (dilution 1:5000, Chemicon) and goat-anti-mouse IgG (dilution 1:5000, Millipore, Temecula, CA, USA) served as secondary antibodies. The membranes were incubated for a short period with ECL detection reagent (ECL™, Amersham/GE Healthcare, München, Germany). Finally, the proteins were visualized using the Fusion FX7 system (Peqlab, Erlangen, Germany). β-actin (dilution 1:1000, Sigma, Taufenkirchen, Germany) served as the internal control. The visualized protein bands were analyzed using ImageJ software. The relative amount of protein was calculated in relation to the β-actin expression. Untreated but ischemia/reperfused hepatocytes served as positive controls.

### Statistical analysis

Data are presented as mean ± SEM. Statistical analysis was performed with Prism 6 software (Graph Pad) using one-way Anova and T-Test as appropriate. Differences with p<0.05 were considered statistically significant.

## Results

### Metabolic activity of human hepatocytes increases after levosimendan treatment and IRI

**Fig 1:**
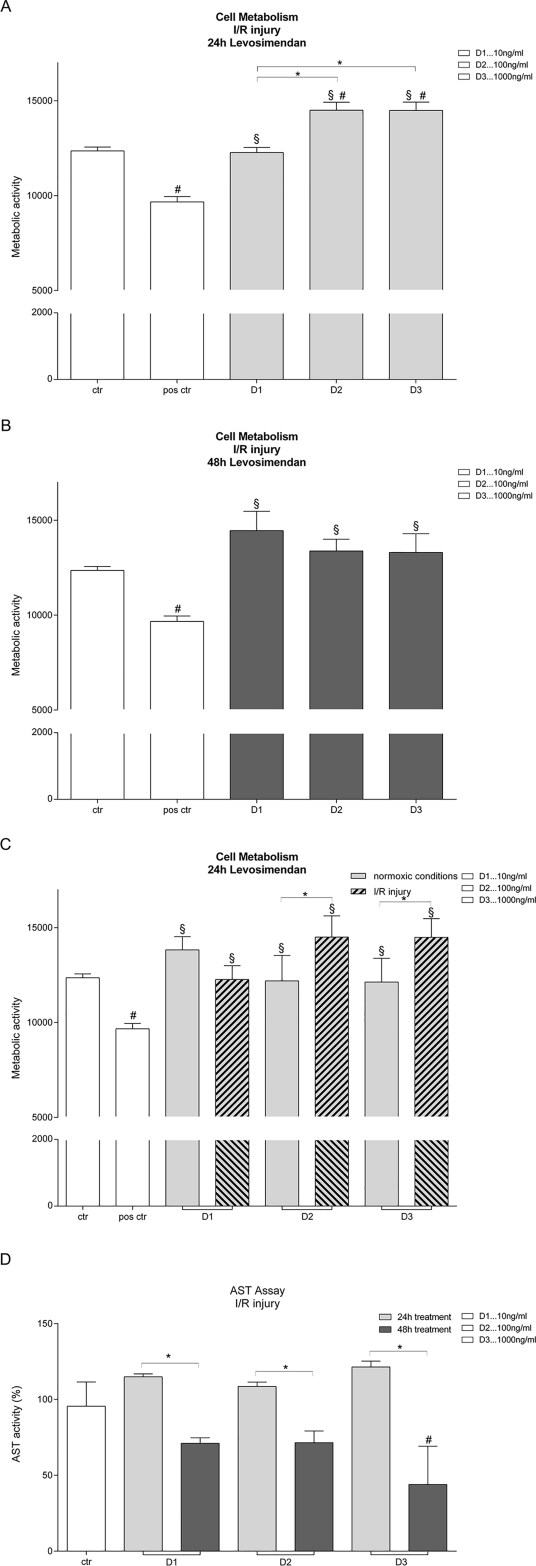
Metabolic activity and AST levels of human hepatocytes after treatment with levosimendan. **(A)** The metabolic activity of human hepatocytes increased after 24 hour treatment with levosimendan after I/R injury in a dose-dependent manner. **(B)** After a 48 hour treatment with levosimendan after I/R injury, the metabolic activity is increased but there is no dependency detectable. **(C)** Normoxic and ischemic/reperfused human hepatocytes were compared regarding metabolic activity after a 24 hour treatment with levosimendan. After I/R the protective effect of levosimendan is amplified compared to normoxic groups. **(D)** AST levels of ischemic/reperfused hepatocytes are significantly reduced after 48 hour treatment with levosimendan compared to 24h treatment. Human hepatocytes are treated with 10ng/ml (D1), 100 ng/ml (D2) and 1000ng/ml (D3) levosimendan. Saponin served as positive control. Untreated but ischemia/reperfused hepatocytes served as control. (each experiment n = 3), # p<0.05 vs ctr., § p<0.05 vs pos. ctr., * p<0,05.

Levosimendan treatment for 24h during IRI increased the metabolic activity of human hepatocytes compared to control. This increase was dose-dependent, starting with 10ng/ml levosimendan and reaching its peak at 1000ng/ml levosimendan (Fig 1A). 48h levosimendan treatment also significantly increased the overall metabolic activity of hepatocytes. Prolonged treatment did not yield any difference with regard to levosimendan dosage (Fig 1B). Comparison between 24h levosimendan treatment with 10ng/ml, 100ng/ml and 1000ng/ml in normoxic versus IRI hepatocytes showed a significant increase in cellular viability in the IRI group(Fig 1C).

Examination of AST as a marker of hepatic injury showed (Fig 1D) showed that the reduction in AST levels was significantly greater after 48h than after 24h levosimendan treatment following IRI.

### Anti-apoptotic effects elicited by levosimendan during normoxic and hypoxic conditions

**Fig 2:**
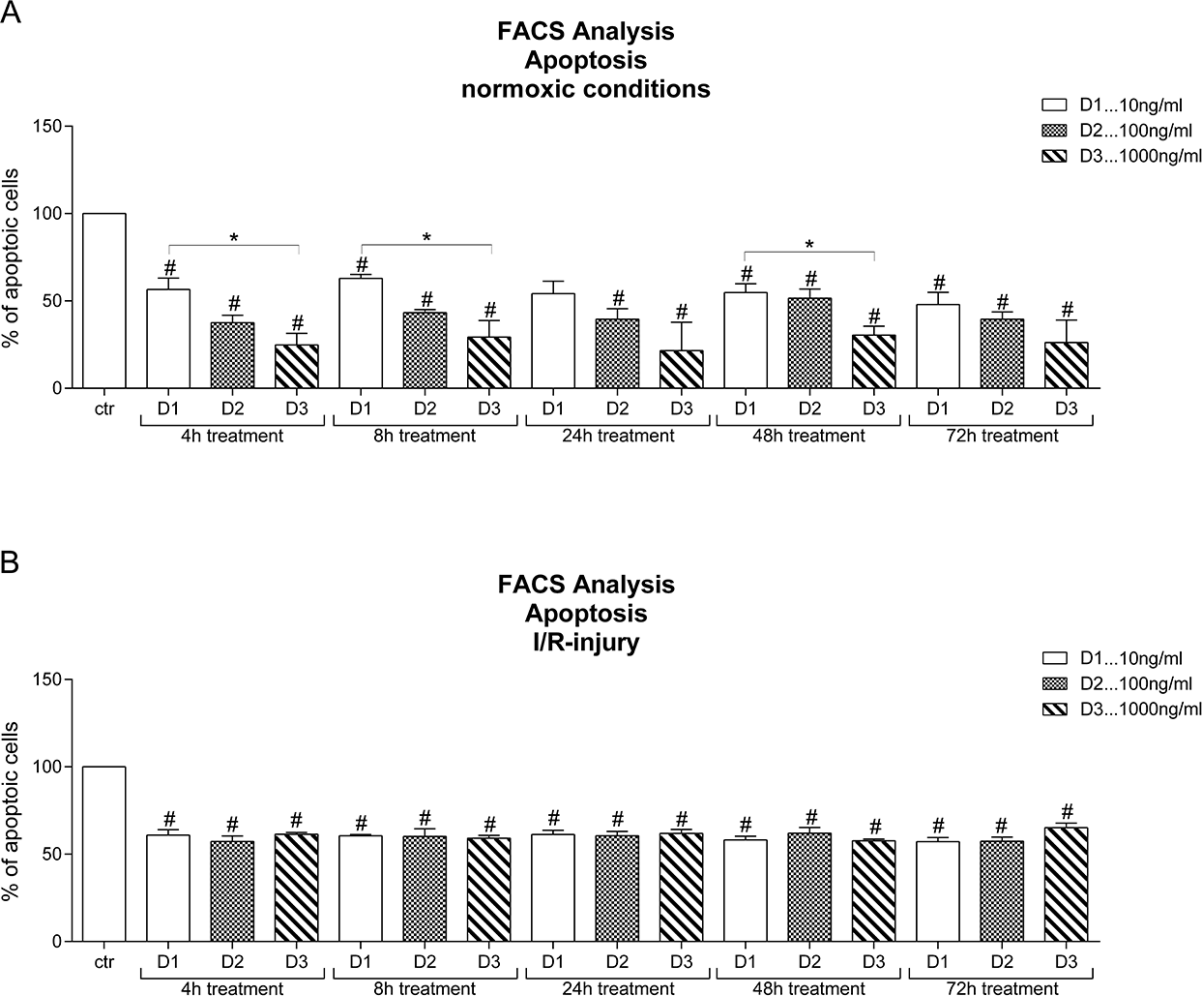
Total number of apoptotic hepatocytes after treatment with levosimendan. **(A)** The number of apoptotic hepatocytes treated under normoxic conditions is reduced in a dose dependent manner compared to the untreated normoxic control group. **(B)** Under I/R conditions, the number of apoptotic hepatocytes after treatment with levosimendan is reduced compared to the untreated ischemia/reperfused control group, but there is no dose-dependency detectable. Human hepatocytes are treated with 10ng/ml (D1), 100 ng/ml (D2) and 1000ng/ml (D3) levosimendan. (each experiment n = 3–4), # p<0.05 vs ctr., § p<0.05 vs pos. ctr., * p<0,05.

The evaluation of hepatocytes treated with levosimendan showed that the number of apoptotic (early and late) hepatocytes was significantly lower in all groups than in the control group (Fig 2A). Furthermore, a dose dependent difference between 10ng/ml, 100ng/ml and 1000ng/ml levosimendan was observed at almost all time points in normoxic hepatocytes exposed to levosimendan treatment, with no statistically significant difference but a trend towards a greater effect yielded by higher levosimendan dosages observed after 24h and 72h treatment.

The number of apoptotic hepatocytes after IRI and levosimendan treatment significantly dropped in all groups compared to control (Fig 2B). A dose-dependency was not seen in any of the IRI groups.

Additionally, normoxic and IRI hepatocytes treated with levosimendan were evaluated with a special focus on early apoptosis (data not shown). The treatment with 10ng/ml and 100ng/ml levosimendan significantly reduced the number of early apoptotic hepatocytes after IRI in comparison with the respective normoxic groups. This effect was observed at all time points (4h, 8h, 24h, 48h and 72h) but was reversed in both normoxic and IRI hepatocytes when treated with 1000ng/ml levosimendan (S2 Fig).

### Effects of levosimendan on BAX and Bcl-2 levels after liver IRI

**Fig 3:**
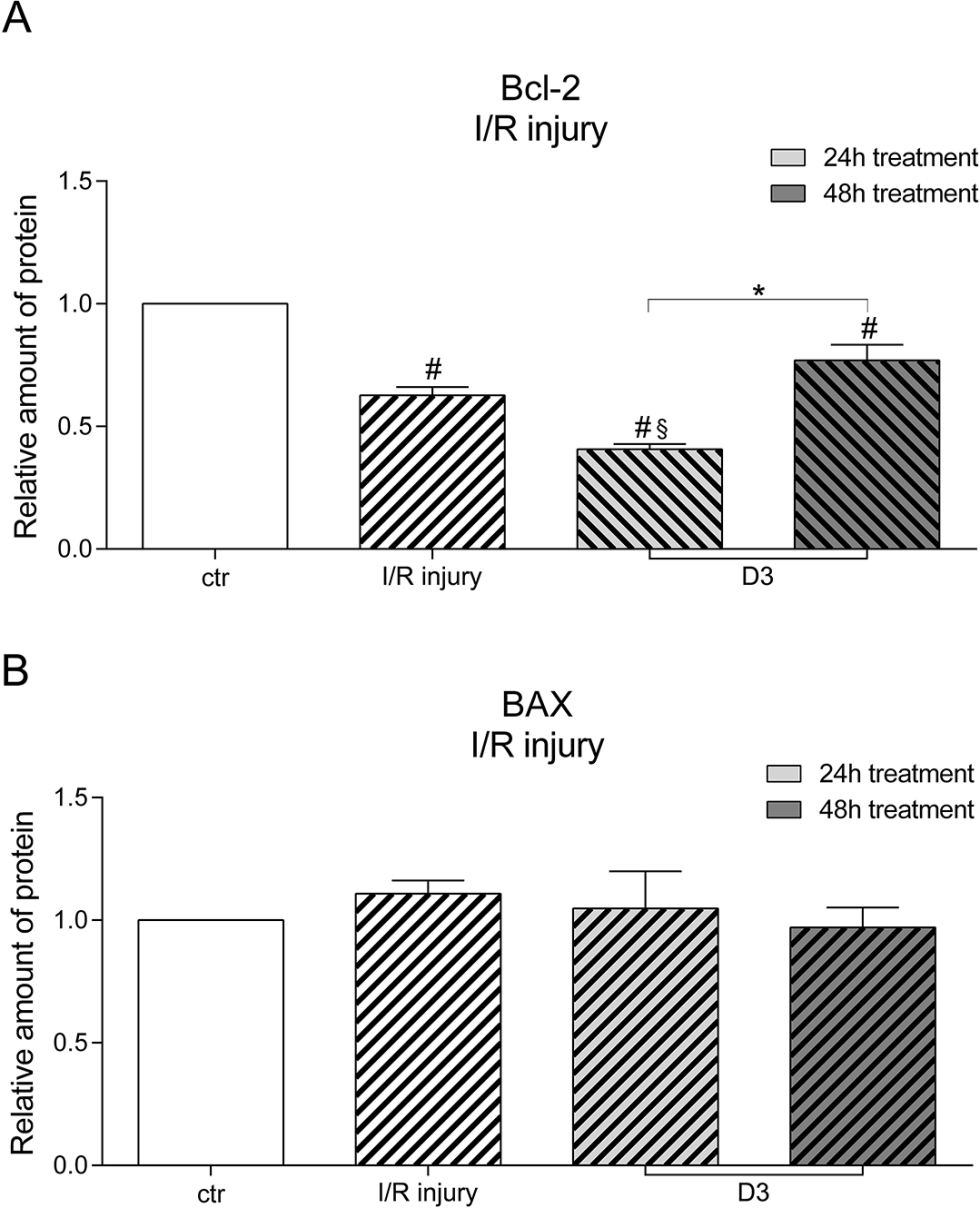
Relative amount of Bcl-2 and BAX after treatment with levosimendan. **(A)** Under hypoxic conditions treatment with levosimendan significantly increases the amount of Bcl-2 in a time-dependent manner. **(B)** Under hypoxic conditions the amount of BAX does not significantly decrease compared to ischemic/reperfused hepatocytes. Human hepatocytes are treated with 1000ng/ml (D3) levosimendan. Untreated but ischemia/reperfused hepatocytes served as positive control. (each experiment n = 2–3), # p<0.05 vs ctr., § p<0.05 vs pos. ctr., * p<0,05.

24h and 48h treatment with 1000ng/ml levosimendan during reperfusion significantly increased the relative amount of the anti-apoptotic protein Bcl-2 in a time-dependent manner (Fig 3A). However, high dosages and long term treatment did not achieve a significant increase in Bcl-2 levels compared to IRI hepatocytes without levosimendan treatment. Human hepatocytes treated with 1000ng/ml levosimendan during reperfusion did not express different levels of the pro-apoptotic protein BAX compared to control (Fig 3B).

The analysis of both proteins in normoxic hepatocytes treated with 1000ng/ml levosimendan showed an increase in Bcl-2 compared to control and a reduction of BAX compared to control. However, the differences determined were not statistically significant (data not shown).

## Discussion

The aim of the present study was to investigate the effects of levosimendan, a mito K(ATP) channel opener [7], on human hepatocytes after IRI. We demonstrated that levosimendan increases metabolic activity and attenuates hepatocyte apoptosis.

IRI is a frequent cause of hepatic failure after transplantation. Within the early stages of reperfusion, microcirculation failure is brought about by endothelial cell swelling [17] and vasoconstriction. Up-regulation of endothelin-1 [18] and down-regulation of nitric oxide (NO) lead to vasoconstriction of the sinusoidal lumen. This in turn reduces leukocyte velocity and enhances leukocyte-endothelial cell contacts. These effects result in leukostasis and prolong the ischemic phase even though reperfusion has already started. Consequently, TNF-α and IL-1, which both play essential roles in IRI, are produced [19] and IL-8 synthesis is induced. IL-8 in its turn increases the expression of adhesion molecules (β-integrins) that promote interaction between leukocytes and sinusoidal endothelial cells and, consequently, enhance leukocyte migration.

Moreover, IRI causes injury to hepatic mitochondria. Permeability transition pores (PTPs), i.e. nonselective pores within the inner mitochondrial membrane [20], are opened during reperfusion and lead to rupture of the outer mitochondrial membrane. The pro-apoptotic factor cytochrome c is released into the cytosol, and apoptosis due to caspase-9 and caspase-3 is activated.

The apoptotic pathway is regulated by the Bcl-2 gene family. Bcl-2 prolongs cell survival by inhibiting apoptosis and reducing hepatic IRI [21]. BAX, in contrast, promotes the opening of PTP [22].

In order to identify the exact mechanisms by which levosimendan exerts its effects in human hepatocytes, several tests were performed. MTT served to evaluate the overall metabolic activity of human hepatocytes after treatment with levosimendan, and we were able to demonstrate that levosimendan in fact increases the metabolic activity of human hepatocytes in a dose-dependent manner. A time-dependent treatment effect, in contrast, could not be shown. The protective effect of levosimendan was amplified in cells previously exposed to IRI. This is in keeping with recent studies in patients with septic shock that suggested positive effects of levosimendan on mitochondrial function [23]. Since mitochondrial integrity is crucial for the metabolic activity of cells, the results of these studies underline our findings. Of note, our results illustrate a protective effect of levosimendan on human hepatocytes especially after IRI.

Based on the experiment outlined above, we chose to analyze AST levels in human hepatocytes. We were able to demonstrate that levosimendan attenuates AST levels in ischemic/reperfused human hepatocytes. Long term treatment, in particular, was found to exert a positive effect on AST levels, which serve as markers of liver damage and are significantly elevated after IRI. Of note, our findings are in keeping with previous studies in animal models that showed AST levels to decrease when levosimendan was administered prior to IRI [24].

In this respect, it is essential to keep in mind that apoptosis represents one of the important pathways involved in IRI [25]. A protective effect based on levosimendan influencing the apoptotic pathway in hepatocytes was, for example, demonstrated in a rat model. [16] We found that treatment with levosimendan under normoxic conditions reduced apoptosis in human hepatocytes and that this effect was particularly pronounced at high levosimendan dosages as proven by the fact that the greatest reduction of apoptosis was determined at all points in time in those cells treated with high levosimendan concentrations. In cell populations exposed to IRI, levosimendan was additionally found to reduce the total number of apoptotic cells in comparison with populations not exposed to levosimendan. This effect, however, was not dose dependent.

It is furthermore noteworthy that levosimendan especially reduces apoptosis in human hepatocytes when they were exposed to IRI. Our findings confirm the results of previous animal studies and supplement them with respect to human hepatocytes. Beyond these findings, our results also demonstrate a protective effect of levosimendan on hepatocytes under normoxic conditions. From this, it may be concluded that levosimendan will, in patients with heart failure, not only increase myocardial contractility but might also exert a protective effect on the liver, which would be of considerable interest as patients with heart failure tend to develop hepatic impairment due to chronic hepatic hypoperfusion.

Western blotting was applied in order to shed light on the mechanisms underlying the effects of levosimendan in terms of apoptosis. The groups that were compared were chosen in keeping with previous tests. The anti-apoptotic protein Bcl-2 and the pro-apoptotic protein BAX were examined. Our findings demonstrate that the Bcl-2 concentration is increased in a dose dependent manner in normoxic cells (data not shown). Under hypoxic conditions, the amount of Bcl-2 is significantly increased in a time dependent manner. However, this effect did not suffice to fully reverse IRI. In addition, the amount of BAX is significantly reduced in a time- and dose dependent manner in normoxic cells treated with levosimendan (data not shown). In cells exposed to IRI and treated with levosimendan, BAX levels were similar to controls and no reduction could be detected. From this, it may be gathered that treatment with levosimendan in this respect is not capable of reversing the effects of IRI.

Down-regulation of BAX and up-regulation of Bcl-2 achieved by treating cells with levosimendan were reported from a number of animal models [15; 26]. While our results also indicate a protective interference of levosimendan with Bcl-2 and BAX levels, the changes achieved in the levels of these apoptotic pathway proteins were not sufficient to fully reverse the damage caused by IRI. These findings highlight that in addition to Bcl-2 and BAX, other mechanisms must be involved in the protective effect of levosimendan in IRI and that the results of animal studies in this respect fail to fully reflect the response to levosimendan treatment in human hepatocytes.

Nevertheless, our findings justify speculations in terms of the possible interactions between the suggested mechanisms of action. Recent studies reported that pharmacological postconditioning might be a new way to protect organs from IRI [27] and suggested that the mechanism underlying these effects may be the opening of mitoK(ATP) channels [28]. In our study, human hepatocytes were exposed to the mitoK(ATP) opener levosimendan during IRI. Hence, the number of apoptotic cells after IRI was significantly reduced when treated with levosimendan compared to control. The opening of mitoK(ATP) channels results in blockage of the mitochondrial permeability transition pore (MPTP) [29], and therefore the loss of membrane potential is prevented and apoptosis due to cytochrome c loss is inhibited. This could be a further explanation why BAX is reduced and Bcl-2 is increased.

Moreover, it could be suggested that levosimendan influences the upstream apoptotic pathway. Interleukin 10 (IL-10) inhibits the production of pro-inflammatory cytokines such as IL-1 and TNF-α. Therefore, the release of cytochrome c is attenuated and caspase-3 activity is reduced. Moreover, IL-10 up-regulates Bcl-2. It could thus be suggested that levosimendan activates IL-10. However, the interplay between these factors should be the object of further studies.

Regarding study design, it could be considered to subject cells to levosimendan treatment before exposing them to ischemia and reperfusion in future studies, thus adding an interesting dimension to the subject of the present study.

The present study provides new insights into the protective effects of levosimendan following IRI in human hepatocytes. Our findings could well have implications in a clinical setting, especially in patients undergoing liver transplantation. With IRI playing a pivotal role in short and long-term rejection of liver grafts [30], levosimendan might offer new ways of attenuating graft dysfunction after liver transplantation.

## Supporting information

S1 Fig Study design. (TIFF)

S2 Fig FACS analysis: Early apoptosis under normoxic conditions vs. I/R injury. Human hepatocytes are treated with 10 ng/ml (D1) 100 ng/ml (D2) and 1000 ng/ml (D3) levosimendan.
(TIFF)

## Abbreviations

AST: aspartate aminotransferase
ATP: adenosine triphosphate
BAX: BCL2 associated X
Bcl- 2: B-cell lymphoma 2
FACS: fluorescence-activated cell sorting
I/R injury; IRI: ischemia/reperfusion injury
MnSOD: manganese-dependent superoxide dismutase
MTT: 3-(4,5-dimethylthiazol-2-yl)-2,5-diphenyltetrazolium bromide
NO: nitric oxide
PTP: permeability transition pore
