# Supplementary figures and images for: Levosimendan protects human hepatocytes from ischemia-reperfusion injury
Source: PLoS One. 2017 Nov 16;12(11):e0187839. doi: 10.1371/journal.pone.0187839 (PMC5690693; doi:10.1371/journal.pone.0187839)

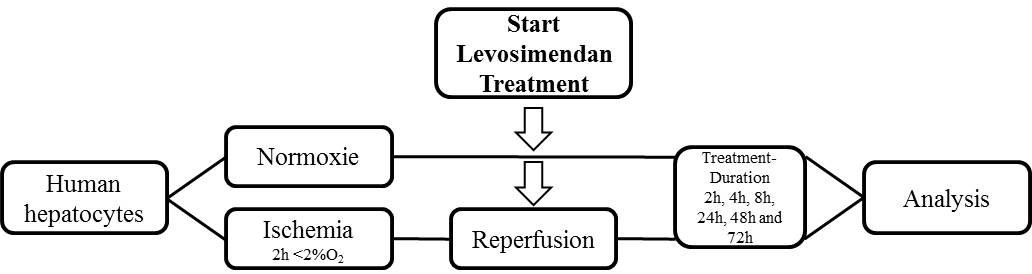

Supplement: S1 Fig — (TIFF) [file pone.0187839.s001.tiff]

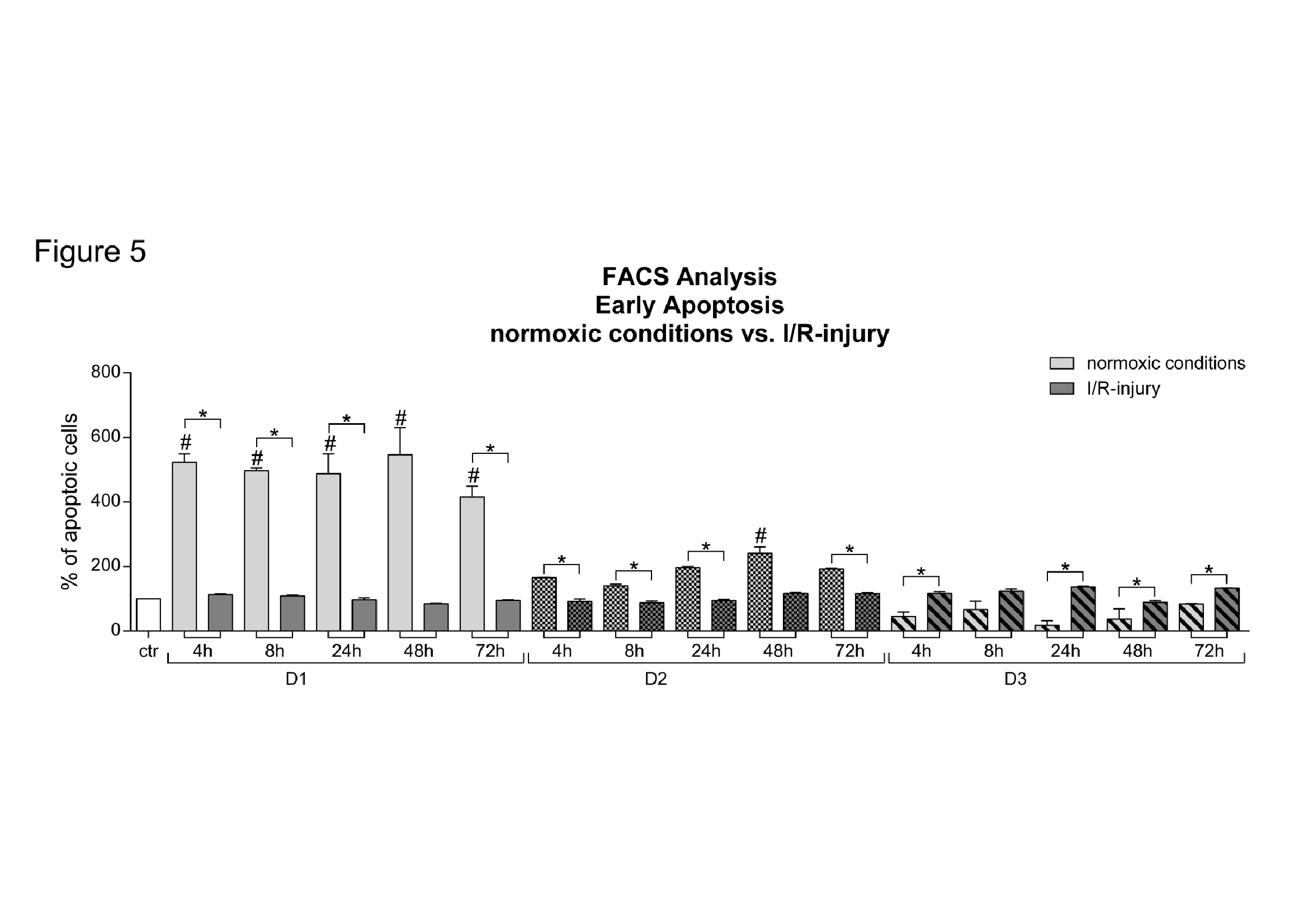

Supplement: S2 Fig — Human hepatocytes are treated with 10 ng/ml (D1) 100 ng/ml (D2) and 1000 ng/ml (D3) levosimendan. (TIFF) [file pone.0187839.s002.tiff]
